# Age-related declines in muscle and respiratory function are proportionate to declines in performance in Master Track Cyclists

**DOI:** 10.1007/s00421-021-04803-4

**Published:** 2021-09-13

**Authors:** Pablo Duro Ocana, Mohammad Z. Darabseh, Kengo Ishihara, Aseel Aburub, Fabio Zambolin, Gallin Montgomery, Richard Mills, Matteo Scorcelletti, James Cameron, Bergita Ganse, Hans Degens, Liam Bagley

**Affiliations:** 1 Department of Life Sciences, Centre of Musculoskeletal Sciences and Sport Medicine, Manchester Metropolitan University, John Dalton Building; Chester Street, Manchester, M1 5GD UK; 2 Department of Food Sciences and Human Nutrition, Faculty of Agriculture, Ryukoku University, Shiga, 520-2194 Japan; 3 School of Allied Health Professions, Keele University, Staffordshire, ST5 5BG UK; 4 Department of Sport and Exercise Sciences, Centre of Musculoskeletal Sciences and Sport Medicine, Manchester Metropolitan University, All Saints Building; Oxford Rd, Manchester, M15 6BW UK; 5 Department of Health Professions, Manchester Metropolitan University, Cavendish Building; Cavendish Street, Manchester, M1 6BG UK; 6 Department of Surgery, Innovative Implant Development, Saarland University, Kirrberger Str. 1, 66421 Homburg, Germany; 7 Institute of Sport Science and Innovations, Lithuanian Sports University, Kaunas, Lithuania

**Keywords:** Master athletes, Lung function, Respiratory function, Maximum respiratory pressure, Muscle function, Muscle architecture, Ageing, Track cycling

## Abstract

**Purpose:**

Respiratory and musculoskeletal function decline with age, irrespective of physical activity levels. Previous work has suggested that the age-related rate of decline in function of these two systems might be similar, but it is not known to what extent each system contributes to decreasing performance in ageing master cyclists. Therefore, the purposes of this study are (1) whether the age-related rate of decline in respiratory function, respiratory muscle strength, muscle architecture, muscle function, haemoglobin concentration, haematocrit and performance in master cyclists is uniform and (2) which parameters contribute most to the reduction in performance with age.

**Methods:**

Master cyclists were recruited during the Track Cycling Masters World Championship 2019 in Manchester. Respiratory function and respiratory muscle strength were determined using spirometry and a mouth pressure device, respectively. Muscle architecture was determined using ultrasonography, and muscle function by countermovement jump.

**Results:**

Forced expiratory volume in the first second, forced vital capacity, fascicle length, muscle thickness, take-off velocity, jump power, jump power per body mass, handgrip strength, haemoglobin concentration and performance correlated negatively with age (*p* ≤ 0.043). The age-related rate of decline did not differ significantly between parameters (*p* = 0.124), but it was slower for haemoglobin concentration (*p* = 0.041). Take-off velocity was the major determinant of performance in 200, 500 and 2000 m track cycling disciplines (*R*^2^_adj_ = 0.675, 0.786 and 0.769, respectively; *p* < 0.001).

**Conclusion:**

Age-related decline in respiratory and muscle system is accompanied by a similar rate of decline in performance. The major contribution to the age-related decline of performance is reduced muscle function, specifically take-off velocity.

## Introduction

Regular physical activity is widely considered the best intervention to protect against the age-related decrement in overall fitness (McPhee et al. 2016). Indeed, the major factor of overall fitness reduction is the decline of physical activity with age (Leyk et al. 2010). Disuse and low levels of physical activity have been shown to lead to muscle atrophy (Degens and Alway 2006) and reduced ventilatory function (George et al. 2014). Even though physical activity helps to maintain high levels of fitness through age, ageing is a progressive process that affects muscle and respiratory fitness (McKendry et al. 2018). This is also reflected by the progressive decline in both pulmonary function, aerobic capacity and maximal peak power generation during the lifespan (Roman et al. 2016; Pearson et al. 2002).

The decrease in power-generating capacity is accompanied by a reduction in muscle size in both, athletes (Couppé et al. 2014) and non-athletes (McPhee et al. 2018; Couppé et al. 2014). Likewise, the respiratory muscle strength, vital capacity and other parameters of maximal ventilation show similar age-related rate of decline in athletes and non-athletes (Degens et al. 2013; Harik-Khan et al. 1998; McClaran et al. 1985). It is therefore, not surprising that there is a close correlation between respiratory function and skeletal muscle index (Sawaya et al. 2018), and between maximal inspiratory pressure (MIP), maximal expiratory pressure (MEP) and handgrip strength (Efstathiou et al. 2016). In a previous cross-sectional study, we saw that anaerobic and aerobic power showed a proportional age-related rate of decline, as reflected by a constant aerobic:anaerobic power ratio throughout life (Bagley et al. 2019).

Concomitant with these changes in muscle and respiratory function is the progressive reduction in track cycling performance (world records) from middle-aged to advanced age (Union Cycliste Internationale 2021). While this provides circumstantial evidence that the age-related rate of decline in respiratory and skeletal muscle function leads to a proportional reduction in cycling performance, no studies have systematically investigated this. Taken together, it appears that related declines in physical function and performance may be due to a stochastic accumulation of (micro) damage over time (Degens 2012) that affects different systems of the human body similarly. Despite some evidence pointing toward a similar age-related decline in muscle and respiratory function, evidence is limited from studies that have analysed the decline of both systems in the same population.

Even though the physiological systems are interrelated, the age-related decline of some parameters may play a more important role than others in the reduction of specific sport performances with age. For instance, the main determinant of performance in endurance disciplines is VO_2_max (Tanaka and Seals 2003), while muscle function is the main determinant in power disciplines (Pearson et al. 2002). Cycling performance depends primarily on a combination of power output, and cardiovascular and pulmonary capacities (Phillips and Hopkins 2020), but the importance of each factor is also likely to differ between distances cycled. For example, in cycling sprint performance, the shortening velocity of the muscle is likely to be an important factor of performance, with an increasingly important contribution of aerobic power with increasing cycling distances (Martin et al. 2007). The velocity of contraction is not only determined by the contractile properties of the muscle, but also the force-generating capacity, or size of the muscle (Degens 2019). Indeed, the size of the Vastus Laterallis (VL) and Vastus Medialis muscles have been reported to be major determinants of 6 s cycling sprint performance (Martin et al. 2007). Further supporting this notion is the observation that the combination of lean thigh volume and pedalling rate accounted for 83% of the variability in power production ability across life in cycling sprint performance, where thigh muscle volume accounted for 76% of that variability (Martin et al. 2000). This impact of muscle mass on muscle functionality, and ultimately in performance, are also observed in recreationally active adults where the principal determinant of walking speed was found to be take-off velocity (*V*_off_) in a counter-movement jump (Maden-Wilkinson et al. 2015).

While expiratory flow limitations and diaphragm fatigue can be detrimental to exercise performance in endurance athletes (Dempsey et al. 2008), it is unlikely to have a significant impact on the sprint performance of track cyclists. This can, however, not be entirely excluded, as an inverse relationship between spirometry parameters and sprint performance has been reported (Bhatt et al. 2015).

Given the previously observed age-related decrements in respiratory and musculoskeletal function, and performance and the suggestion that ageing is a reflection of a stochastic accumulation of micro damage, it is hypothesized that cycling performance, skeletal muscle and respiratory function show a proportional age-related rate of decline. Due to the short duration of track cycling races, it is hypothesised that muscle architecture and function are the major determinants of track cycling performance.

## Materials and methods

### Study design

Master cyclists (60 men; (38–84 years) and 15 women; (34–80 years)) were recruited during the UCI Track Cycling World Masters Championships 2019 (Manchester, United Kingdom). Participants with chronic respiratory complaints, cardiovascular, neuromuscular, or metabolic disease, or those who had a leg fracture in the past two years were excluded from the study. The study was approved by the Science and Engineering Research Ethics Committee at Manchester Metropolitan University. All participants provided written informed consent before participating, conforming to the Declaration of Helsinki.

### Participant characteristics

Height (in m) and body mass (in kg) were assessed using a stadiometer and digital scales, respectively. The haemoglobin (Hb) concentration (Hemocue, Ängelholm, Sweden) and haematocrit (Hct) were measured from capillary blood obtained by finger prick. Training hours per week were self-reported by participants. Age-graded performance was calculated as the performance in the best event (prime event) of a person of a given age as a percentage of the world record at that age. Participant characteristics are summarized in Table 1.

**Table 1:** Participant characteristics

	Men	Women	*p* value
Age (years)	61 ± 11 (60)	50 ± 12 (15)	0.002*
Height (m)	1.75 ± 0.07 (55)	1.68 ± 0.05 (15)	< 0.001*
Body mass (kg)	81.5 ± 13.8 (55)	65.9 ± 7.7 (15)	< 0.001*
BMI (kg/m^2^)	26.6 ± 3.6 (55)	23.2 ± 2.4 (15)	0.004*
[Hb] (g/L)	141 ± 17 (51)	135 ± 8 (15)	0.327
Hct (%)	44.9 ± 4.1 (50)	42.5 ± 3.4 (14)	0.035*
Hb/Hct	3.15 ± 0.37 (50)	3.20 ± 0.34 (14)	0.755
Hours of training per week	11.2 ± 4.8 (47)	9.7 ± 3.0 (14)	0.281
Age-graded performance (%)	90.7 [86.5–94.5] (55)	92.26 [88.7–95.8] (13)	0.274
200 m time (s)	13.0 [12.4–13.5] (30)	13.0 [12.7–14.6] (5)	0.552
500 m time (s)	40.6 [38.8–42.7] (32)	42.3 [40.3–45.3] (8)	0.153
2000 m time (s)	158 [150–168] (25)	160 [156–174] (10)	0.321

All data are presented as mean ± SD (*N*) or median [IQR] (*N*)

Age-graded performance represents the median values of the prime event time of each participant normalised to the world record of the prime event at the, respective, category—age. 200, 500 and 2000 m time represent the median race times of all participants that competed on the respective discipline, independently of their prime event

*BMI* Body mass index; *Hb* Haemoglobin; *Hct* Haematocrit

^*^p value < 0.05 denotes significant sex effect

### Muscle architecture

Muscle architecture was analysed using Real-time B-mode ultrasonography with a linear-array probe (VF13-5, Siemens ACUSON P500, Erlangen, Germany). Muscle thickness (MT, in mm), fascicle length (*L*_f_, in mm) and pennation angle (in degrees) of the VL muscle were determined at 50% of the femur length (McPhee et al. 2018), while the cyclist was seated with the knee and hip at 90° of flexion. All ultrasound scanning and image analysis (Image J, v1.8.0_112; National Institutes of Health, Bethesda, MA) was completed by the same investigator. All architectural parameters were given as the average of three measurements in the image. MT was defined as the shortest distance between the superficial and deep aponeuroses. *L*_f_ was calculated using the extrapolation method (Brennan et al. 2017) when necessary. The pennation angle was defined as the angle between the deep aponeurosis and the fascicles. The intra-rater coefficient of variance (CV) for all the measurements was below 10% (CV MT = 1%, CV *L*_f_ = 6%, CV pennation angle = 9%).

### Muscle function: jump power and handgrip strength

Handgrip strength and jump power were collected as measurements of muscle function. Participants performed three countermovement jumps (CMJ) (Bagley et al. 2019) with the hands placed on the hip, on a force platform (Leonardo Mechanograph^®^: Novotec Medical GmbH, Pforzheim, Germany). The jump with the highest power output (in kW), was selected for final analysis. Jump power and jump power per kg of body mass was computed by the system (Leonardo Mechanography v4.4 Software^®^: Novotec Medical GmbH, Pforzheim, Germany). *V*_off_ (in m·s^−1^) was calculated as:

$${V}_{\mathrm{off }}= g \times \left(\frac{{t}_{\mathrm{air}}}{2}\right)$$

where *g* is gravitational acceleration (9.81 m·s^−2^), and *t*_*air*_ represents the flight time (s) (Degens et al. 2019).

Handgrip strength (in kg) was measured with a hand-held dynamometer (Takei Handgrip, Hab International Ltd. Warwickshire, United Kingdom) as an indicator of overall muscle strength (Stark et al. 2011). The elbow was placed at the side of the body and flexed at 90^0^, with the wrist in neutral position.

### Respiratory function (spirometry)

Respiratory function was measured by spirometry. All spirometry measurements were performed using a Micro Medical Spiro USB Spirometer and analysed with Spida 5 software (Cardinal Health, UK). The spirometer was calibrated daily according to the ATS/ERS recommendations (Miller et al. 2005). The participants were seated with feet rested flat on the floor during the test. The participants were instructed to inhale forcefully and maximally followed by forceful exhalation while wearing a nose clip. Three successful procedures were required to conclude the test, with a maximum of 8 manoeuvres conducted to achieve 3 successful procedures. According to the ATS/ERS recommendation, a procedure was considered successful if there was a plateau in the exhalation, exhalation lasted > 6 s, there were no coughs and between-test variations for Forced Vital Capacity (FVC,) and Forced Expiratory Volume in the first second (FEV_1_) were < 0.15 L. The measured parameters were: FEV_1_, FVC, FEV_1_/FVC ratio and Peak Expiratory Flow (PEF). FVC and FEV_1_ are reported in L and PEF in L/min. The predicted values for FEV_1_ (FEV_1pred_), FVC (FVC_pred_) and PEF (PEF_pred_) were calculated as previously described (Degens et al. 2013) according to equations that include age and height as provided by The Third National Health and Nutrition Examination Survey taking into account ethnic background (Hankinson et al. 1999). Spirometer flow diagrams were examined for participants to ensure that participants were performing proper manoeuvres. The lower limit of normal (LLN) for FEV_1_/FVC and FEV_1_ were also calculated using the equations presented in Hankinson et al. (1999). Any participants with an FEV_1_/FVC and FEV_1_ < LLN were excluded from the analysis.

### Respiratory pressure

The maximal inspiratory (MIP) and expiratory (MEP) pressures were measured using a portable mouth pressure device (MicroRPM, Cardinal Healthcare, UK) as estimators of respiratory muscle strength. Participants were instructed to inhale or exhale as forcefully as possible after total exhalation or inhalation, respectively, into the portable MicroRPM (Hautmann et al. 2000; The American Thoracic Society 2002). To measure the maximal sniff nasal inspiratory pressure (SNIP), participants were instructed to place a probe in one of their nostrils while the other nostril was closed and then inspired as fast and as forceful as possible via the nose. For all manoeuvres, attempts were repeated, with a 30-s interval between each attempt to prevent the development of respiratory muscle fatigue, until the maximum value was achieved. Data are reported in centimetre of water (cm H_2_O).

### Age-related rate of decline in muscle, respiratory function and cycling performance

The age-related rate of decline in muscle architecture, handgrip strength, jump power, respiratory function, maximum respiratory pressures and performance was calculated as the annual percentage change from the predicted value at the age of 35 years. The predicted value at the age of 35 years was calculated for each sex separately, from the regression equation of each parameter against age.

### Discipline performance

Times of individual 200, 500 and 2000 m disciplines for each participant were collected from the results book of the 2019 Master Track Cycling World Championships (Union Cycliste Internationale 2021). Performance was considered as the time of a participant in a given discipline as a percentage of the predicted time for a master cyclist of 35 years old. The predicted time was estimated from the linear regression between the times of all participants in a discipline and the age of the participants for each sex separately. Some participants competed in more than one event, and in that case the event with the best performance was considered their prime event and selected for analysis. The performances in 750 and 3000 m were not included in the analyses as only 35- to 49-year-old people competed in these events.

### Statistical analysis

Statistical analyses were performed using SPSS software (IBM Corporation, NY, US). Data were assessed for normality with the Shapiro–Wilk test. To assess differences between sexes, a multivariate ANOVA was performed with sex as factor. If the data were not normally distributed, a non-parametric test (Mann–Whitney test) was performed. An ANCOVA with sex and the different parameters as factors, and age as covariate was used to assess differences in the age-related rate of decline of the different parameters. Multiple regression analysis between the times of the different disciplines as independent variable, and age, sex, body mass, muscle architecture, jump power, respiratory function and respiratory pressures as dependent variables, was performed to assess the determinants of performance in each discipline. Further multiple regression analysis with the major determinant of performance was carried out to better understand what determines performance.

## Results

Upon calculating the participants LLN for FEV_1_/FVC, eleven participants had < LLN of FEV_1_/FVC. One man participant was excluded after checking the spirometer flow diagrams. For the FEV_1_ LLN, the remaining 10 participants had FEV_1_ > LLN and therefore, no further participants were excluded from the analysis.

### Comparison between sexes

Table 1 shows participant characteristics. The average age of the men was higher than that of the women (*p* = 0.002). Men were taller than women (*p* < 0.001) and their body mass and BMI were higher than that of women (*p* ≤ 0.004). Hct was 5.0% higher in men than women (*p* = 0.035), but there was no significant difference in [Hb] between the sexes (*p* = 0.327). Table 2 shows the respiratory function, respiratory pressures, muscle architecture, jump power and handgrip strength of the participants. Men had a higher MT (13.5%), jump power (25.3%) and handgrip strength (7.0%) than women (*p* ≤ 0.001), but there were no significant differences in fascicle length, pennation angle, take-off velocity and jump power per kg of body mass between men and women (*p* = 0.460, *p* = 0.322, *p* = 0.068, *p* = 0.091, respectively). Table 2 shows also that FEV_1_ (25.3%), FEV_1_pred% (6.7%), FVC (14.3%) PEF (28.5%), MIP (21.1%) and MEP (17.8%) were higher in men than women (*p* ≤ 0.040). Age-graded performance and weekly hours of training were similar between sexes in the different disciplines (*p* = 0.274 and *p* = 0.281, respectively) (Table 1).

**Table 2:** Muscle architecture, muscle function, spirometry and respiratory pressure parameters

	Men	Women	P value
MT (mm)	28.9 ± 4.2 (56)	25.0 ± 3.0 (15)	0.001*
*L*_f_ (mm)	140 ± 31 (55)	137 ± 19 (15)	0.460
Penation angle (°)	11.7 ± 1.7 (55)	11.0 ± 1.7 (15)	0.322
Jump power (kW)	3.40 ± 0.86 (55)	2.54 ± 0.59 (14)	0.001*
Jump power/kg body mass (W/kg)	41.9 ± 8.4 (55)	38.3 ± 7.3 (14)	0.091
*V*_off_ (m·s^−1^)	2.35 ± 0.31 (55)	2.17 ± 0.33 (14)	0.068
Hand grip strength (kg)	47.4 ± 9.1 (45)	34.6 ± 5.4 (15)	< 0.001*
FEV_1_ (L)	4.00 ± 0.66 (45)	2.99 ± 0.66 (14)	0.010*
FEV_1pred%_	104 ± 15 (45)	97 ± 15 (14)	0.057
FVC (L)	4.9 ± 0.90 (45)	4.2 ± 1.2 (14)	0.040*
FVC_pred%_	106 ± 15 (45)	104 ± 12 (14)	0.398
FEV_1_/FVC	73.0 ± 7.7 (45)	72.4 ± 12.7 (14)	0.850
PEF (L/min)	530 ± 136 (45)	379 ± 115 (14)	< 0.001*
PEF_pred%_	104 ± 24 (45)	97 ± 9.5 (14)	0.406
MIP (cmH_2_O)	110 ± 28 (48)	87 ± 28 (14)	0.004*
MEP (cm H_2_O)	158 ± 36 (48)	130 ± 20 (14)	0.009*
SNIP (cmH_2_O)	103 ± 38 (48)	108 ± 22 (14)	0.588

All data are presented as mean ± SD (*N*)

Spirometry predicted values were calculated from the equations of the software provided by the spirometer

*MT* Muscle thickness; *L*_*f*_ Fascicle length; *V*_*off*_ Take-off velocity; *FEV*_*1*_ Forced expiratory volume in one second; *FVC* Forced vital capacity; *PEF* Peak expiratory flow; *MIP* Maximal inspiratory pressure; *MEP* Maximal expiratory pressure; *SNIP* Sniff nasal inspiratory pressure; *L* Litre; *cmH*_*2*_*O* centimetre of water

^*^p value < 0.05 denotes significant sex effect

### Age-related rate of decline in muscle, respiratory function, blood and performance

FEV_1_, FVC, PEF, MIP, MEP, *L*_f_, MT, *V*_off_, jump power, jump power per kg of body mass, handgrip strength, [Hb] and performance correlated negatively with age (*p* < 0.05), while there were no significant correlations between pennation angle, SNIP and Hct with age (Table 3). Sex had no significant effect on the age-related rate of decline in any of the parameters (*p* = 0.985). The ANCOVA analysis showed significant differences in the age-related rate of decline of the different parameters (*p* = 0.032), with the [Hb] showing the lowest age-related rate of decline (Table 3). An additional ANCOVA analysis excluding [Hb] showed no significant difference in the age-related rate of decline between parameters (*p* = 0.102).

**Table 3:** Age-related rate of decline

	*N*	Age-related rate of decline	*R* value	*p* value
*L*_f_	70	6.5%	−0.455	< 0.001*
MT	71	5.1%	−0.507	< 0.001*
Pennation angle	70	1.8%	−0.169	0.169
*V*_off_	66	6.9%	−0.744	< 0.001*
Jump power	69	10.2%	−0.626	< 0.001*
Jump power/kg body mass	69	8.9%	−0.677	< 0.001*
Handgrip strength	60	7.3%	−0.565	< 0.001*
FEV_1_	59	7.1%	−0.608	< 0.001*
FVC	59	6.0%	−0.450	< 0.001*
FEV_1_/FVC	59	1.2%	−0.232	0.077
PEF	59	4.6%	−0.295	0.023*
MEP	62	4.1%	−0.354	0.005*
MIP	62	4.8%	−0.260	0.043*
SNIP	62	5.4%	−0.176	0.176
Hb	66	2.6%	−0.320	0.009*
Hct	64	1.1%	−0.179	0.161
Performance	58	5.9%	−0.451	< 0.001*

All data are presented as mean ± SD (*N*)

*L*_*f*_ fascicle length; *MT* muscle thickness; *FEV*_*1*_ forced expiratory volume in one second; *FVC* forced vital capacity; *PEF* peak expiratory flow; *MIP* maximal inspiratory pressure; *MEP* maximal expiratory pressure; *SNIP* sniff nasal inspiratory pressure; *Hb* haemoglobin; *Hct* haematocrit

^*^p value < 0.05 denotes significant decline with age

### Determinants of track cycling performance

The major determinant of 200, 500 and 2000 m performance was *V*_off_ (*R*^2^_adj_ = 0.690, 0.794 and 0.769, respectively; *p* < 0.001). There was an additional contribution of PEF in 200 m performance (*R*^2^_adj_ = 0.765; *p* = 0.019). *R*^2^_adj_ increased to 0.844 in 500 m performance after adding pennation angle to the model (*p* < 0.001) and 0.906 after adding MT (*p* < 0.001). *R*^2^_adj_ increased to 0.823 in 2000 m performance after adding to the model pennation angle (*p* < 0.001) and 0.855 after adding age (*R*^2^_adj_ = 0.855, *p* < 0.001). The major determinant of *V*_off_ was MT (*R*^2^_adj_ = 0.409; *p* < 0.001). *R*^2^_adj_ increased to 0.591 after adding age to the model (*p* < 0.001) and 0.654 after adding sex (*p* < 0.001).

## Discussion

The main observation of the present study is that respiratory function, muscle architecture, muscle function and cycling performance showed proportional rates of annual decline that was similar in men and women. This suggests that ageing is uniform between physiological systems. In addition, we observed that the take-off velocity during a countermovement jump was the main determinant of cycling performance, irrespective of sex and age.

### Interrelation between the age-related rate of decline of peripheral muscle, respiratory function and respiratory muscles

**Fig. 1:**
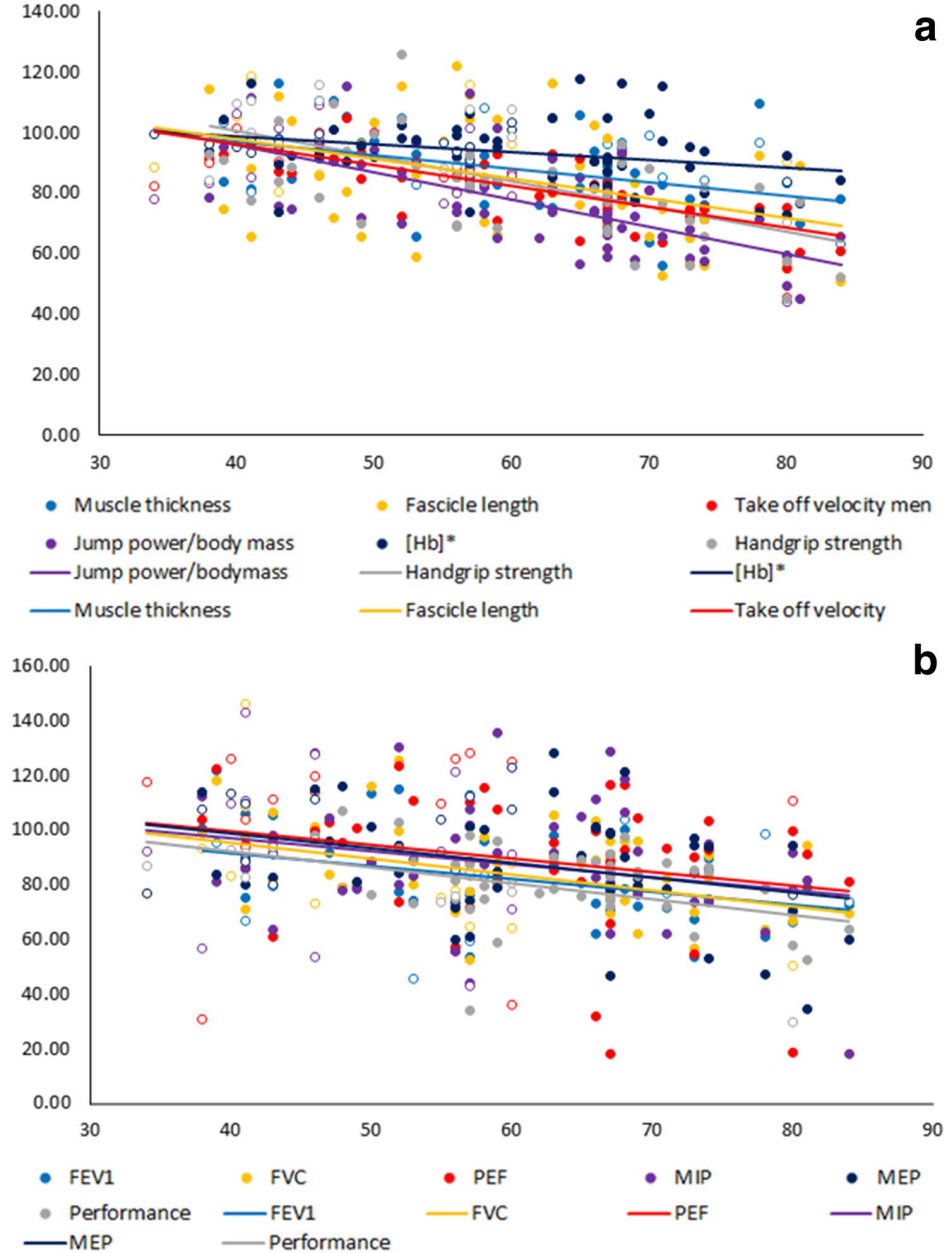
**a** Age-related rate of decline in muscle architecture, jump power per kg of body mass, handgrip strength and haemoglobin concentration [Hb] as a proportion of the value at the age of 35 years. Closed circles represent men and open circles women. *p-value < 0.05 denotes significant difference in the age-related decline**. b** Age-related rate of  decline in respiratory function, respiratory pressure, and performance as a proportion of the value at age 35 years. Closed circles represent men and open circles women

It has been reported that the age-related rate of decline in muscle function, respiratory function and respiratory muscle strength does not differ significantly between athletes and non-athletes (Degens et al. 2013; Pearson et al. 2002). Here, we extend these observations and show that respiratory function, respiratory muscle strength, MT, *L*_f_ and jump power in cycling athletes decline at similar rates with age. These changes were accompanied with a proportional decline in cycling performance (Table 3, Fig. 1). This suggests that ageing proportionately affects different physiological systems, regardless of the physical activity levels or the type of training practiced (Bagley et al. 2019; Degens et al. 2013; Michaelis et al. 2008; Pearson et al. 2002). Indeed, ageing affects the performance in different athletic disciplines similarly (Ganse et al. 2018) and is seen in athletics, swimmers and even chess players (Berthelot et al. 2011) and suggests that ageing affects all human systems similarly. The uniformity in the decline of different physiological parameters and performance suggests that there is a common cause of the detrimental changes in all sorts of organ system during ageing, which may well be a stochastic accumulation of (micro) damage over time (Degens 2012).

### Respiratory function and respiratory muscle strength

Similar to previous observations in non-athletes, endurance and power athletes (Degens et al. 2013), we observed here a significant age-related rate of decline in FEV_1_, FVC, PEF, MIP and MEP, irrespective of sex. Even though pulmonary function may be better in master athletes than age-matched non-athletes (Degens et al. 2013; Hagberg et al. 1988), which may be explicable by selection where men and women with better pulmonary function remain active, they still suffer from an age-related reduction in pulmonary function. Indeed, in this cohort of track cyclists, predictive values of respiratory function are around 100%, supporting the notion that physical activity does not protect against the age-related decline in pulmonary function (Roman et al. 2016; Saltin and Grimby 1968). Part of the decline in spirometry may be attributable to the loss of respiratory muscle strength (Degens et al. 2013), but also other factors, such as decrements in lung recoil and chest compliance (Lesauskaite and Ebejer 1999), will contribute to an age-related reduction in ventilator capacity.

### Muscle architecture and muscle function

Similar to previous observations in non-athletes (Narici et al. 2003), we found that MT declined by 0.51% per year, which was accompanied by an annual 0.65% decline in *L*_f_. This indicates that even in master cyclists, ageing leads into a reduction of the number of sarcomeres in series. As resistance training-induced hypertrophy is accompanied by an increase (Erskine et al. 2011; Reeves et al. 2004) and the disuse-induced atrophy by a decrease (Narici et al. 2002) in pennation angle. It is perhaps somewhat surprising that we did not find an age-related decrease in pennation angle. It should be noted, however, that MT—the product of *L*_f_ and the sine of the pennation angle—is fully explicable by a decrease in *L*_f_. The implication of these changes is that the force-generating capacity is not affected, but the shortening velocity of the muscle, and hence, the ability to generate power—product of force and velocity—is reduced (Degens et al. 2009). This impaired power-generating capacity during ageing may be further aggravated by the selective atrophy of type II fibres (Barnouin et al. 2017) and slowing of type I fibres, even in athletes (Korhonen et al. 2006). Such a reduced shortening velocity may have functional consequences, as in non-athletes the age-related reduction in *V*_off_ during a CMJ explained 30–40% of the variation of walking speed (Maden-Wilkinson et al. 2015). Therefore, with age, muscle quality apart from muscle mass, may be an important contributor to the decline of muscle function with age (Goodpaster et al. 2006).

### Determinants of track cycling performance

**Fig. 2:**
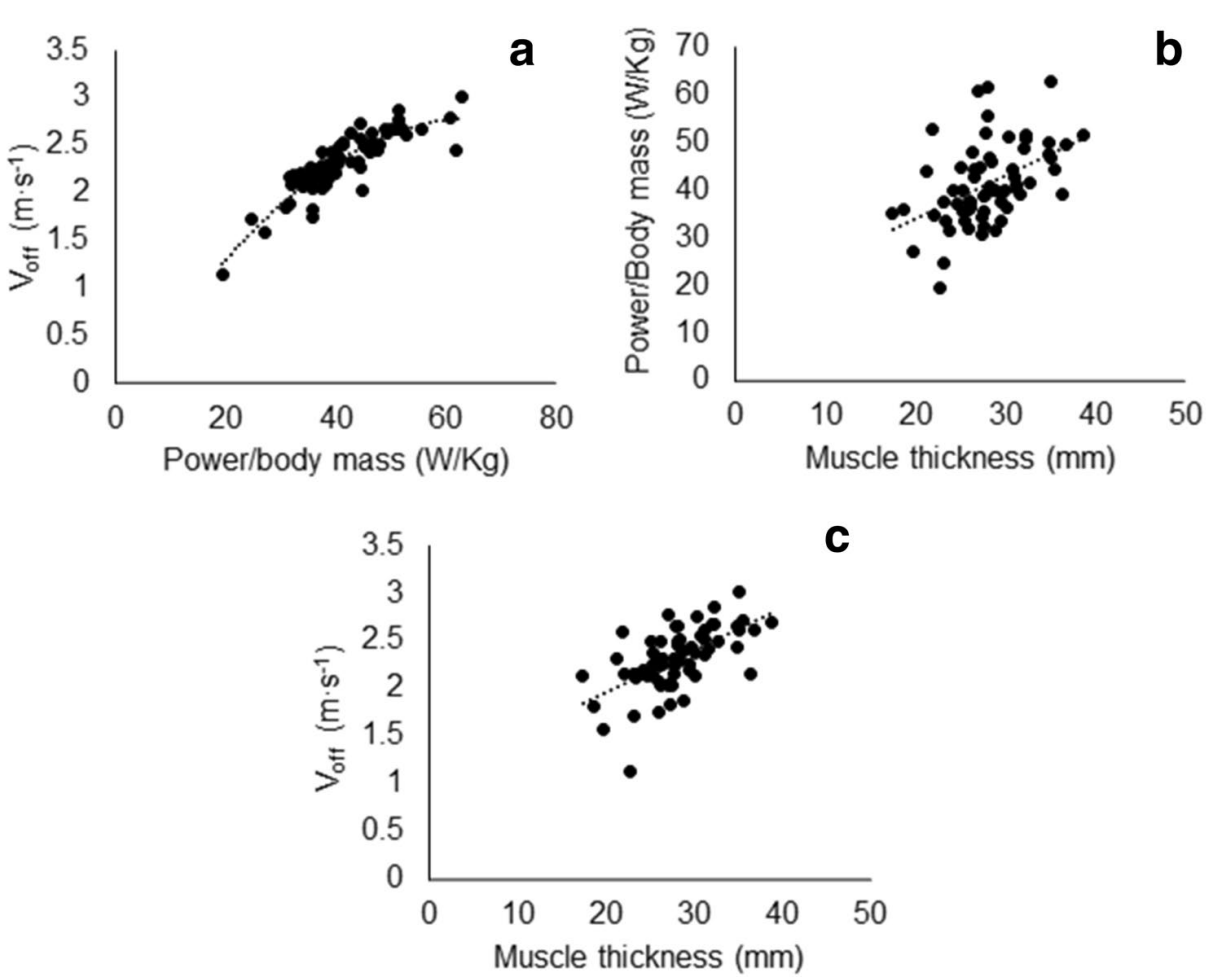
**a** Relationship between Voff (take-off velocity) and power per kg of body mass: *R*^2^ = 0.7822, *p* < 0.001**. b** Between muscle thickness and power per kg of body mass: *R*^2^ = 0.2275, *p* < 0.001**. c** Between muscle thickness and Voff: *R*^2^ = 0.3577, *p* < 0.001

Here, we found that *V*_off_ was the main determinant of cycling performance in ≤ 2000 m events, irrespective of age and sex. While this may indicate a slowing of the muscle contractile properties, it may be explicable by a diminished force generation ability, which would cause the muscles to work on a slower part of the force–velocity relationship (Degens 2019). Unfortunately, we did not directly measure the force-generating capacity of the muscle, but in support of this, we found that MT, an indirect measure of force-generating capacity (Muraki et al. 2013), was the major determinant of *V*_off_ and correlated with the power-generating ability of the master cyclists (Fig. 2). Future studies should address whether indeed there is an age-related slowing in muscle contractile properties and/or, a diminished force-generating capacity that caused this age-related decline in *V*_off_ during a countermovement jump and concomitant decline in cycling performance.

## Limitations

Although the women were on average younger than the men, when split by age and sex into 15-year groups, physiological parameters remained significantly different (*p* < 0.05) between sexes. The relatively low number of women in the study may cause weaker correlations in the women than the men, but even so, the data of the women overlap those of the men in Fig. 1. This indicates that even though the number of women was low, the pattern emerges that men and women show a similar age-related decline. No control group was included in this study, perhaps making it difficult to assess whether track cycling attenuates the age-related decline in the function of different physiological functions. However, the %predicted values of the spirometry parameters may indicate that regular track cycling does not attenuate the age-related decline in pulmonary function (Table 2). However, the cross-sectional nature of the study somewhat limits this assumption and further longitudinal study is necessary to confirm this finding. Other studies have shown that the %decline in jump power (Michaelis et al. 2008) and VO_2max_ (Tanaka and Seals 2008) is similar in athletes and non-athletes, suggesting that regular exercise does not attenuate the ageing process per se (Degens 2012).

## Conclusion

It was found that the uniform decline in peripheral muscle architecture and function, respiratory function and respiratory muscle strength with age, in master cyclists, was accompanied by a proportional rate of decline in performance. It appeared that *V*_off_ was the major determinant of performance (at least in ≤ 2000 m events), irrespective of age and sex. It remains to be seen whether this decline in take-off velocity during a countermovement jump (*V*_off_) is attributable to a slowing of the contractile properties and/or a loss of force-generating capacity of the muscle. However, our observation that muscle thickness, a proxy for force-generating capacity, correlated with *V*_off_ suggests that the decline in force is the main cause of the decline in *V*_off_, rather than slower contractile properties.

## Data Availability

The datasets generated and/or analysed during the current study are available from the corresponding author on reasonable request.

## Abbreviations

CV: Coefficient of variance
FVC: Force vital capacity
FVC_pred_: Force vital capacity predictive values
FEV_1_: Force expiratory volume during the first second
FEV_1 pred_: Force expiratory volume during the first second predictive values
g: Gravity
Hb: Haemoglobin
Hct: Haematocrit
L_f_: Fascicle length
MEP: Maximal expiratory pressure
MIP: Maximal inspiratory pressure
MT: Muscle thickness
PEF: Peak expiratory flow
PEF_pred_: Peak expiratory flow predictive values
SNIP: Nasal inspiratory pressure
T_air_: Time on air
VL: Vastus lateralis
V_off_: Take-off velocity
